# Improvement of Mechanical Properties and Electrical Resistivity in Giant Magnetostrictive Tb-Dy-Fe Alloy via Co-Addition of Al and Si Elements

**DOI:** 10.3390/ma19010154

**Published:** 2026-01-01

**Authors:** Qianhao Zhu, Jiawang Cheng, Jiheng Li, Xing Mu, Xiaoqian Bao, Jie Zhu, Xuexu Gao

**Affiliations:** 1 State Key Laboratory for Advanced Metals and Materials, University of Science and Technology Beijing, Beijing 100083, Chinachengjw0102@163.com (J.C.); gaox@skl.ustb.edu.cn (X.G.); 2 Department of Functional Material Research, Central Iron and Steel Research Institute Co., Ltd., Beijing 100081, China; muxinghalo@126.com

**Keywords:** Tb-Dy-Fe alloy, element addition, magnetostriction, electrical resistivity, mechanical property

## Abstract

Giant magnetostrictive Tb-Dy-Fe alloys are extensively applied in transducers, actuators, and smart sensors owing to their exceptional magnetostrictive response. Nevertheless, in addition to the fracture failure caused by the inherent brittleness of the Laves intermetallic compound, Tb-Dy-Fe alloys also suffer from severe eddy current losses due to low electrical resistivity, both of which limit the practical application of Tb-Dy-Fe alloys. To further enhance the overall performance of Tb-Dy-Fe alloys and expand their application scope, it has become essential to develop materials that exhibit high magnetostrictive properties, high electrical resistivity and excellent mechanical properties simultaneously. In this work, the effects of Al and Si co-addition on the microstructure and multifunctional properties of directionally solidified Tb_0.27_Dy_0.73_(Fe_0.9_Al_0.075_Si_0.025_)_1.95_ (hereafter TDF-AlSi) alloy were systematically investigated. Microstructural characterization revealed that Al partially substitutes Fe atoms in the matrix phase while promoting Al(Tb,Dy)Fe_2_ nanocluster, whereas Si preferentially segregated to grain boundary regions forming Tb_2_Si_3_ and TbSi_1.75_ phases. The bending strength of TDF-AlSi alloy was improved from 43 MPa to 65 MPa, an increase of 51.2%, which was attributed to solid solution strengthening by Al and grain boundary reinforcement by Si-rich precipitates. Meanwhile TDF-AlSi alloy exhibits a 2.4 times increase in electrical resistivity (1.619 μΩ·m), resulting in a 49% reduction of total loss at 1000 Hz. The enhancement of electrical resistivity mainly originated from the lattice distortion induced electron scattering by Al substitution and electron impedance at grain boundaries via silicide precipitation. Accompanied by enhancement of mechanical property and electrical resistivity, TDF-AlSi alloy maintained a high magnetostriction strain of 1212 ppm (200 kA/m, 10 MPa pre-compressive stress). The findings of the present study offer valuable theoretical and experimental insights with regard to the optimization of the performance of magnetostrictive Tb-Dy-Fe alloys.

## 1. Introduction

As functional smart materials, magnetostrictive materials will undergo deformation under magnetic fields and change magnetization state under applied stress, thus enabling mutual conversion between mechanical and magnetic energy. Over the past decades, considerable efforts have been devoted to developing high-performance magnetostrictive materials, including Tb-Dy-Fe alloys [1,2], Fe-Ga alloys [3,4,5], Fe-Co alloys [6], and MnCoSi-based alloys [7]. Tb-Dy-Fe alloys have been used in transducers, precision actuators and smart sensors thanks to their giant magnetostrictive coefficients, high energy density and excellent electromechanical conversion efficiency [8]. However, as intermetallic compounds, Tb-Dy-Fe alloys suffer from two critical performance deficiencies: low electrical resistivity (approximately 0.6 μΩ·m) [9] and poor mechanical properties [10,11]. Low resistivity leads to prominent eddy current losses in alternating magnetic fields, resulting in Joule heating and subsequent temperature elevation within the materials. This not only affects the magnetostrictive performance but also reduces the operational stability in devices. Furthermore, the inherent brittleness of C15-type Laves phase impeded mechanical processing, consequently restricting the engineering applicability of these alloys [12].

To address these issues, alloying modification has emerged as an effective approach for improving the comprehensive performance of Tb-Dy-Fe alloys [13,14,15,16]. The introduction of new elements into alloys can significantly alter the original electronic configuration, thereby effectively enhancing the material’s resistivity. Simultaneously, the addition of new elements promoted the precipitation of new phases within the alloy, which plays a crucial role in suppressing crack propagation and can significantly improve mechanical properties. Over recent decades, extensive research efforts have been devoted to exploring the influence of third-element additions on the multifunctional characteristics of Tb-Dy-Fe systems. A variety of transition metals and metalloid elements—including B, Al, Ga, Si, V, Zr, Mn, Co, Ni, Ti, and Cu—have been evaluated as partial substitutes for Fe at the B-site of the RFe_2_ Laves structure [13,14,15,16,17,18,19,20,21,22,23,24]. While these compositional modifications have demonstrated effectiveness in enhancing certain target properties such as mechanical strength or electrical resistivity, they invariably accompany a deterioration in magnetostrictive output to various extents. Among these studies, regarding the issues of mechanical properties and electrical resistivity, Prajapati K. et al. [21] investigated Al substitution for Fe and demonstrated that moderate Al addition had a limited impact on the magnetostrictive properties. A magnetic field of 120 kA/m and pre-compressive stress of 12 MPa caused the magnetostrictive strain of Tb-Dy-Fe(Al) to remain at 1000 ppm. A 10% Al substitution increased the resistivity of Tb-Dy-Fe(Al) alloys by over 50%, reducing eddy current losses. Furthermore, the compressive strength reached 735 MPa, representing an increase of over 120% compared to oriented polycrystalline Tb-Dy-Fe alloys. This demonstrated that Al addition effectively enhances both the resistivity and mechanical properties of Tb-Dy-Fe alloys. The enhancement of resistivity can be attributed to electron scattering caused by lattice distortion when Al atoms substitute Fe atoms in the Laves phase structure [25,26]. The improvement in mechanical properties originates from solid solution strengthening, where the atomic size mismatch between Al and Fe creates local strain fields that impede dislocation motion [26,27]. Xu L.H. et al. [18,28] investigated the effects of Si alloying on Tb_0.3_Dy_0.7_(Fe_1−x_Si_x_)_1.95_ (x = 0, 0.025, 0.1) alloys. Results showed alloying did not alter the MgCu2-type cubic Laves phase structure. When x = 0.025, the magnetostrictive performance reached 1545 ppm under 5 MPa pre-compressive stress and high magnetic field of 4000 Oe, with resistivity enhanced to 1.06 μΩ·m. Microstructural characterization revealed that Si tended to segregate at grain boundaries after alloying. Bodnar W et al. [29] investigated the effects of Mn addition on the magnetic properties and resistivity of Tb_0.27_Dy_0.73_(Mn_1−x_Fe_x_)_2_ alloys. Their results showed that the alloys kept the MgCu_2_-type C15 structure after adding Mn, and the alloy resistivity increased with the amount of Mn. When Mn replaced all of Fe (Tb_0.27_Dy_0.73_Mn_2_), the total resistivity at room temperature reached about 1.3 μΩ·m. Therefore, achieving a balance between magnetostriction performance and other properties like mechanics and resistivity, while enhancing overall performance, remains challenging.

In our previous work [30], we investigated the effects of single Al or Si addition on the resistivity and magnetostriction of Tb-Dy-Fe alloys. However, the mechanical properties were not systematically addressed, and the synergistic effects of Al-Si co-addition remained unexplored. To address these limitations, unlike prior studies focused on single-element alloying for specific property enhancement, this work employs Al-Si co-addition to simultaneously regulate both the matrix and grain boundary phases. Specifically, Al is expected to enhance mechanical properties through solid-solution strengthening in the matrix, while Si preferentially segregates at grain boundary regions to form high-resistivity silicides that disrupt intergranular electron transport pathways. This dual-phase modification strategy aims to synergistically improve electrical resistivity and mechanical strength while maintaining acceptable magnetostrictive performance.

## 2. Materials and Methods

Alloys with nominal compositions of Tb_0.27_Dy_0.73_Fe_1.95_ (hereafter TDF) and TDF-AlSi were prepared by vacuum induction melting from high-purity starting metals. To compensate for the volatilization losses of rare earth elements during the melting process, 2–3 wt.% excess Dy and Tb were added. The starting materials were melted under argon protection and held for 3–5 min to ensure compositional homogeneity. Subsequently, the oriented crystals were prepared using directional solidification equipment with a withdrawal rate of 4 mm/min. The temperature gradient at the solid–liquid interface was maintained at approximately 100 K/cm, corresponding to a cooling rate of 7 K/s. The directional solidification was conducted under argon atmosphere to obtain <110> preferentially oriented rods. All property measurements (bending strength, electrical resistivity) were performed on at least three samples for each composition, and the data presented represent the average values.

Crystallographic texture and phase identification were conducted on a Rigaku SmartLab diffractometer with Cu Kα radiation (λ = 0.154 nm), scanning from 10° to 90° (2θ) at 5°/min. Microstructural observations employed multiple techniques: a field-emission scanning electron microscope (FE-SEM, SUPRA™55, Carl Zeiss AG Baden-Württemberg, Germany) for morphological imaging, an electron probe microanalyzer (EPMA-1720, Shimadzu, Tokyo, Japan) for compositional mapping, and a field-emission transmission electron microscope (FE-TEM, FEI Tecnai G2 F30 S-TWIN, 300 kV, 0.14 nm resolution) for high-resolution structural analysis. Electrical resistivity was acquired from rectangular specimens (2 × 2 × 40 mm) via an AT515 precision resistance tester (Applent, Changzhou, China, accuracy: 0.1 μΩ). Magnetostrictive strain measurements utilized resistance strain gauges coupled with a JDAW-2011 system, while magnetic hysteresis behavior was recorded on a NIM-3000S soft magnetic measurement platform. Mechanical properties were assessed through three-point bending tests conducted on an MTS-810 universal testing machine (MTS Systems Corporation, Eden Prairie, MN, USA).

## 3. Results and Discussion

### 3.1. Microstructure

**Figure 1:**
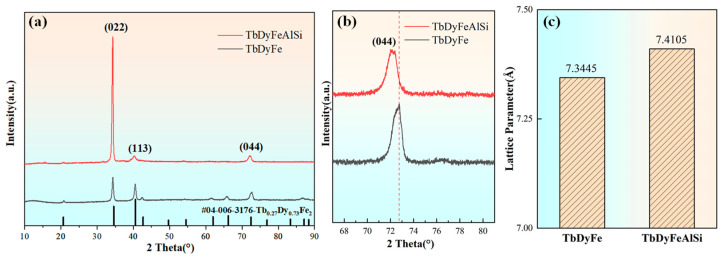
X-ray diffraction analysis of directionally solidified TDF and TDF-AlSi alloys: (**a**) full-range diffraction patterns, (**b**) locally magnified spectrum of the (044) peak, (**c**) lattice parameters calculated from (044) peak positions using Bragg’s law.

Figure 1 shows the XRD patterns of the TDF and TDF-AlSi alloys. The alloy maintains the MgCu_2_-type cubic Laves phase structure after the addition of Al and Si (Figure 1a). Compared to PDF standard cards (#04-006-3176), the directionally solidified samples exhibit significant <110> axial preferred orientation, as evidenced by the highest intensity of the diffraction peak for the (022) plane. In the magnified spectrum in Figure 1b, the (044) peak shifts toward lower angles after alloying, indicating that the lattice parameter changes after Al and Si co-addition. The calculation reveals that the lattice parameter increases from 7.3445 Å for TDF to 7.4105 Å for TDF-AlSi, as shown in Figure 1c, indicating a lattice expansion of approximately 0.9% after alloying. Additionally, the enhanced relative intensity of (022) peak after alloying of Al and Si elements suggests that the composite addition promoted the development of <110> preferred orientation. This enhanced <110> texture may be attributed to modifications in surface formation energy induced by Al-Si co-addition. An analogous phenomenon has been reported in magnetostrictive Fe-Ga alloys, where first-principles calculations demonstrated that B doping altered the surface formation energy and consequently shifted the preferred growth direction from <110> to <100> [31].

**Figure 2:**
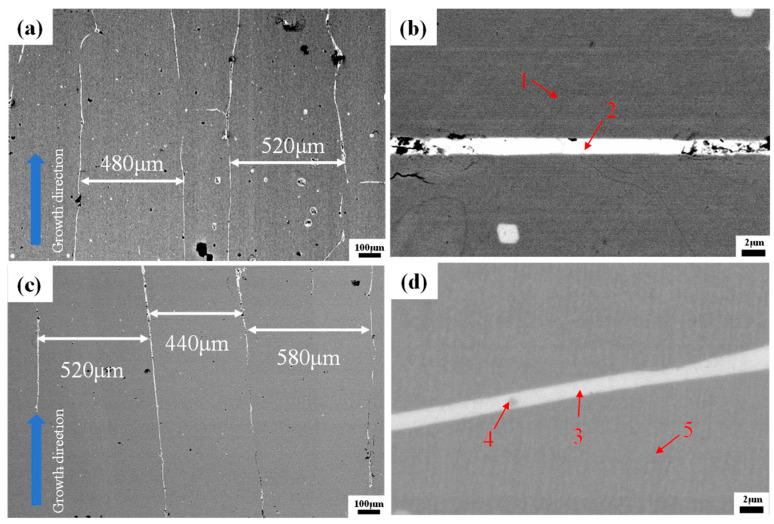
BSE images of directionally solidified <110>-oriented Tb-Dy-Fe alloys: (**a**,**b**) TDF alloy at low and high magnification, respectively, (**c**,**d**) TDF-AlSi alloy at low and high magnification, respectively.

BSE imaging of directionally solidified TDF and TDF-AlSi alloys reveals a dual-phase microstructure, as shown in Figure 2. The predominant phase appears as dark-gray contrast corresponding to the Re(Tb,Dy)-Fe matrix, while bright-white regions indicate RE-rich secondary phases localized at grain boundaries (Figure 2a,c). Consistent with the directional solidification process, the matrix develops an elongated columnar morphology extending several hundred micrometers along the growth direction. The RE-rich phase forms continuous intergranular networks with typical widths in the micrometer range.

The compositional variations across different microstructural regions, as marked in Figure 2b,d, were quantified using energy-dispersive X-ray spectroscopy (EDS), with results summarized in Table 1. For the TDF alloy, the grain boundary phase exhibits enrichment in Tb and Dy, whereas Fe content within the matrix region corresponds well with the designed stoichiometry. Upon Al-Si co-addition, a notable redistribution of alloying elements is observed: Al dissolves preferentially into the matrix phase with relatively uniform distribution, while Si demonstrates a pronounced tendency to accumulate at grain boundaries where it combines with Tb and Dy to form RE(Tb,Dy)-Si intermetallic compounds. Only trace amounts of Si remain detectable within the matrix interior.

**Figure 3:**
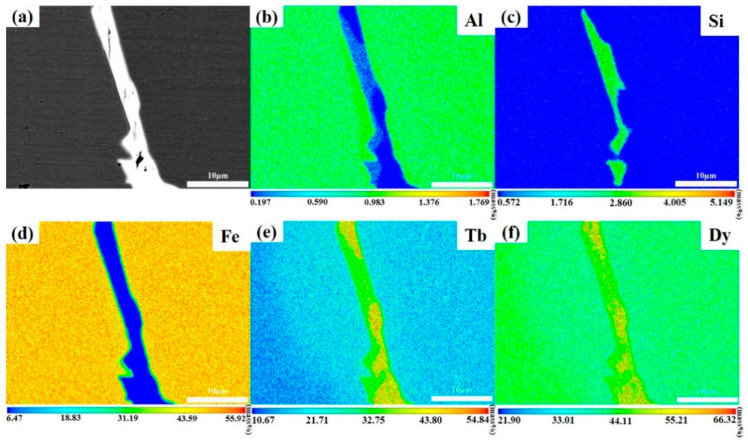
Microstructure and elemental distribution of directionally solidified <110>-oriented TDF-AlSi alloy: (**a**) BSE image, and elemental distribution maps: (**b**) Al, (**c**) Si, (**d**) Fe, (**e**) Tb, and (**f**) Dy.

To further analyze the elemental distribution characteristics in the alloys, the detailed elemental distribution analysis of the TDF-AlSi alloys was performed using an electron probe microanalyzer (EPMA). Figure 3 shows the EPMA results of the grain boundary regions. The BSE image of grain boundary phase is presented in Figure 3a, and Figure 3b–f display the elemental distribution images of Al, Si, Fe, Tb, and Dy, respectively. The elemental distribution analysis reveals the distinct distribution patterns for each element. The element of Al is primarily distributed in the matrix phase region with relatively uniform distribution, while Si exhibits significant enrichment in the grain boundary phase regions, showing the discontinuous distribution characteristics along the grain boundaries. Furthermore, Fe, Tb, and Dy, as the primary alloying elements, are relatively uniformly distributed throughout the matrix phase. These results were consistent with the previous EDS analyses. The distinct distribution behaviors of Al and Si can be attributed to differences in their thermodynamic interactions with the Tb-Dy-Fe matrix. For Al, the atomic radius (0.143 nm) is close to that of Fe (0.124 nm), with a size difference of approximately 15%, which favors the formation of substitutional solid solution. Previous studies on RE(Fe,Al)_2_ Laves phases have demonstrated that Al can substitute Fe atoms while maintaining the C15-type cubic structure [30]. Therefore, Al preferentially dissolves into the (Tb,Dy)Fe_2_ matrix phase and exhibits uniform distribution. In contrast, Si exhibits strong chemical affinity with rare earth elements due to the highly negative formation enthalpy of RE-Si compounds [32]. Consequently, during solidification, Si preferentially segregates to the RE-rich grain boundary regions and reacts with Tb and Dy to form silicide phases.

**Figure 4:**
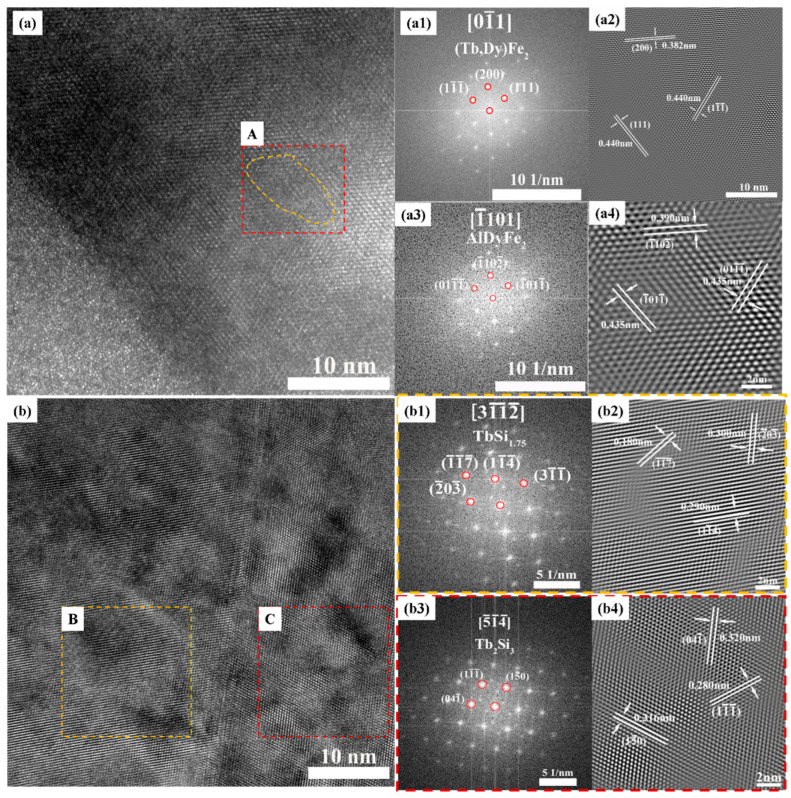
TEM analysis of directionally solidified TDF-AlSi alloy: (**a**) HRTEM image of the matrix phase region, (**a1**) FFT pattern of the overall matrix region shown in (**a**), (**a2**) IFFT image of the overall matrix region, (**a3**) FFT pattern of region A marked in (**a**), (**a4**) IFFT image of region A; (**b**) HRTEM image of the grain boundary region, (**b1**) FFT pattern of region B marked in (**b**), (**b2**) IFFT image of region B marked in (**b**), (**b3**) FFT pattern of region C marked in (**b**), (**b4**) IFFT image of region C marked in (**b**).

The distribution and phase formation of Al and Si elements following composite addition in the <110>-oriented polycrystalline TDF-AlSi alloys was investigated using the focused ion beam (FIB) technique to extract specimens for high-resolution transmission electron microscopy (HRTEM) analysis, as shown in Figure 4. TEM images of the matrix phase and grain boundary phase of the TDF-AlSi alloys are shown in Figure 4a and Figure 4b, respectively.

Figure 4(a1,a3) show the fast Fourier transform (FFT) patterns of the overall matrix phase region and yellow dashed Region A, respectively, with their corresponding inverse fast Fourier transform (IFFT) patterns shown in Figure 4(a2,a4). The analysis of PDF-standard-calibrated interplanar spacing measurements reveals that the FFT pattern of the entire matrix phase region in Figure 4(a1) exhibits high consistency with the DyFe_2_ phase along the [$0\bar{1}1$] zone axis. The interplanar spacing measurements in Figure 4(a2) show $d_{111}=0.440$ nm, $d_{1\bar{1}\bar{1}}=0.440$ nm and $d_{200}=0.382$ nm. Compared with the standard lattice parameters of the DyFe_2_ phase (d_111_ = 0.424 nm, d_200_ = 0.367 nm), the (111) and (200) interplanar spacings increase by approximately 3.8% and 4.1%, respectively. This proportional increase in interplanar spacing indicated that Al predominantly exists in solid solution form within the matrix (Tb,Dy)Fe_2_ phase through partially substituting Fe atoms. Furthermore, an Al-enriched cluster of approximately 10 nm was observed in the matrix phase region (region A in Figure 4a, marked by yellow dashed lines). The combined FFT and EDS (shown in Table 2) analyses identify this cluster as the AlDyFe_2_ phase along the $\left[\bar{1}101\right]$ zone axis, with interplanar spacings $d_{\bar{1}10\bar{2}}$ = 0.390 nm, $d_{\bar{1}01\bar{1}}$ = 0.435 nm and $d_{01\bar{1}\bar{1}}$ = 0.435 nm, as shown in Figure 4(a4), which is consistent with the standard values ($d_{\bar{1}10\bar{2}}$ = 0.394 nm, $d_{\bar{1}01\bar{1}}$ = 0.435 nm and $d_{01\bar{1}\bar{1}}$ = 0.435 nm). Therefore, HRTEM analysis confirms that the element of Al exhibits dual existence forms in the matrix phase region: predominantly as substitutional solid solution atoms replacing Fe positions, and partially as nanoscale Al(Tb,Dy)Fe_2_ precipitate clusters.

The bright-field image in Figure 4b shows distinct phase separation in the grain boundary phase region, forming a complex microstructure with multi-phase coexistence. It can be seen from the FFT images (Figure 4(b1,b3)) that the two regions marked B and C (shown in Figure 4b) correspond to the different phase composition regions within grain boundary phase. TEM analysis of the grain boundary region reveals the complex distribution characteristics of Si elements. Furthermore, the FFT analysis of the region B indicates that it corresponds to TbSi_1.75_ phase along the [$3\bar{11}\bar{2}$] zone axis, with the measured interplanar spacings $d_{\bar{117}}$ = 0.180 nm, $d_{1\bar{1}\bar{4}}$ = 0.290 nm and $d_{\bar{2}0\bar{3}}=$ 0.300 nm, as shown in Figure 4(b2). They are consistent with the standard values ($d_{\bar{117}}$ = 0.181 nm, $d_{1\bar{1}\bar{4}}$ = 0.288 nm and $d_{\bar{2}0\bar{3}}$ = 0.299 nm). Similarly, the FFT analysis of region C shows the correspondence with Tb_2_Si_3_ phase along the $[\bar{514]}$ zone axis, with interplanar spacings $d_{1\bar{11}}$ = 0.280 nm, $d_{04\bar{1}}$ = 0.320 nm and $d_{1\bar{5}0}$ = 0.316 nm, as shown in Figure 4(b4), which are consistent with the standard values ($d_{1\bar{11}}$ = 0.281 nm, $d_{04\bar{1}}$ = 0.322 nm and $d_{1\bar{5}0}$ = 0.316 nm). In summary, the analysis results demonstrate that the element of Si is predominantly enriched in grain boundary phase regions, coexisting as two distinct silicide phases of TbSi_1.75_ and Tb_2_Si_3_. These phases exhibit the intimate interfacial relationships within the grain boundary regions.

To provide a quantitative estimation of the microstructural features described above, the volume fractions of different phases were analyzed. Based on BSE image analysis (Figure 2) and EDS compositional data (Table 1), the volume fractions of different phases were estimated. The RE-rich grain boundary phase (containing silicides) occupies approximately 8–12% of the total volume, as determined by area fraction analysis of BSE images. Within this grain boundary region, the TbSi_1.75_ and Tb_2_Si_3_ silicide phases account for approximately 40–50% of the grain boundary volume based on Si concentration mapping. For the Al(Tb,Dy)Fe_2_ nanoclusters in the matrix phase, TEM observation (Figure 4a) reveals a sparse distribution with an average cluster size of ~10 nm and an estimated volume fraction of approximately 0.1–0.5%, corresponding to a number density of approximately 10^21^–10^22^ m^−3^. While the nanoclusters represent a minor volume fraction, their nanoscale dimensions create substantial interface area (~10^5^–10^6^ m^2^/m^3^) that contributes significantly to electron scattering. The predominant resistivity enhancement originates from the solid solution Al atoms uniformly distributed throughout the matrix phase (~6 at. % based on EDS Point 5 in Table 1), which cause widespread lattice distortion and electron scattering throughout the bulk material.

### 3.2. Mechanical Properties, Resistivity, and Magnetic Properties

The 2 × 2 × 40 mm rectangular specimens were prepared from the TDF and TDF-AlSi alloy samples for electrical resistivity measurements. The electrical resistivity was measured at room temperature using an AT515 resistance tester. The electrical resistivity (ρ) was calculated according to Equation (1):

(1) $$\rho =R\frac{S}{L}$$

where *ρ* is the electrical resistivity in Ω·m; *R* is the resistance in Ω; *L* is the conductor length in m; and *S* is the cross-sectional area of the conductor in m^2^.

**Figure 5:**
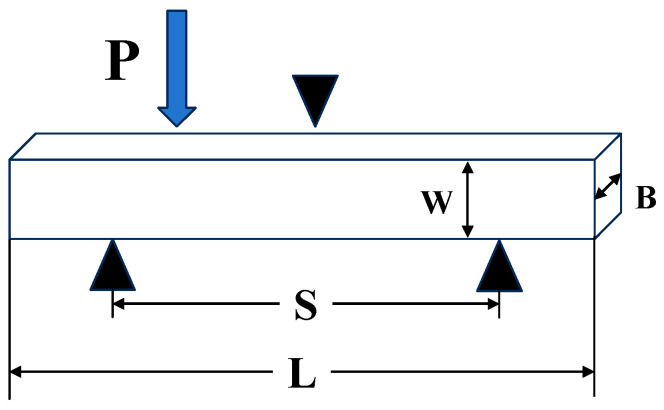
Schematic diagram of three-point bending test specimen.

The bending strengths of TDF and TDF-AlSi alloys were determined using three-point bending tests on an MTS-810 machine at 0.1 mm/min. Specimens measuring 20 × 5 × 2.5 mm were prepared using wire electrical discharge machining with a span length of 16.8 mm. The bending strength was calculated using Equation (2):

(2) $$\sigma =\frac{3PS}{2BW^{2}}$$

where *W* is the specimen thickness; *B* is the specimen width; *P* is the applied load; and *S* is the distance between the two support points. The schematic diagram of three-point bending test specimen is shown in Figure 5.

**Figure 6:**
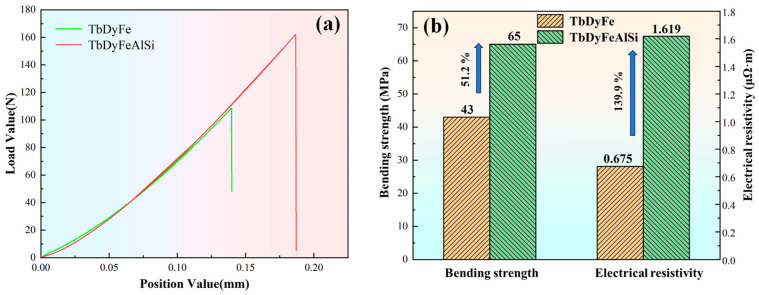
Effect of Al-Si co-addition on bending strength and electrical resistivity of TDF alloy: (**a**) bending test curves, (**b**) comparative analysis of bending strength and electrical resistivity.

Figure 6 demonstrates the significantly enhanced bending strength and electrical resistivity in Al/Si co-added TDF-AlSi alloy relative to the unmodified TDF alloy. The bending strength increases by 51.2% from 43 MPa to 65 MPa, while electrical resistivity rises by 139.9% from 0.675 μΩ m to 1.619 μΩ m. The composite addition of Al and Si elements significantly enhances the bending strength of the TDF alloys. The reasons for the improvement of mechanical properties of TDF-AlSi alloys by adding Al can be summarized through two mechanisms: solid solution strengthening and precipitation strengthening. On the one hand, in the matrix phase, Al partially substituted Fe atom positions to form solid solutions, resulting in the lattice distortion and the enhanced resistance to dislocation movement. On the other hand, the hexagonal Al(Tb,Dy)Fe_2_ phase clusters contribute to the additional precipitation strengthening. In addition, the reason why Si element improves the mechanical properties of the Al-Si co-added Tb-Dy-Fe alloys can be summarized as follows: the element of Si is precipitated as Tb_2_Si_3_ and TbSi_1.75_ phases within grain boundary phase regions, contributing to precipitation strengthening and optimizing the grain boundary structure, thereby improving crack propagation resistance. In summary, these microstructural modifications worked synergistically to significantly enhance the alloys’ bending strength.

According to Matthiessen’s rule (*ρ* = *ρ*_0_ + *ρ_ph_* + *ρ_m_*), the total resistivity consists of residual resistivity (*ρ*_0_), phonon scattering contribution (*ρ_ph_*), and magnetic scattering contribution (*ρ_m_*) [27]. The Al-Si co-addition enhances resistivity through: (1) Increased ρ_0_ due to lattice distortion caused by Al solid solution (lattice parameter change from 7.3445 Å to 7.4105 Å, ~0.9% expansion) and the formation of nanoscale interfaces around Al(Tb,Dy)Fe_2_ clusters; (2) Enhanced *ρ_ph_* resulting from the expanded lattice which modifies the phonon spectrum and increases electron-phonon scattering probability; (3) Increased *ρ_m_* from magnetic scattering at nanocluster-matrix interfaces. The formation of poorly conductive silicide phases (TbSi_1.75_, Tb_2_Si_3_) at grain boundaries further impedes intergranular electron transport. Similar mechanisms have been reported in Mn-doped Tb-Dy-Fe alloys [26].

To semi-quantitatively evaluate the relative contributions to resistivity enhancement, the total resistivity increase (Δ*ρ* = 0.944 μΩ·m) can be decomposed into matrix-dominated (Δ*ρ*_matrix_) and grain-boundary-dominated (Δ*ρ*_GB_) contributions. Considering that Al is uniformly distributed throughout the matrix phase at ~6 at. % (Table 1), while Si-containing silicides occupy only 8–12% of the total volume at grain boundaries, the matrix-phase scattering is expected to dominate. Based on the volume fraction analysis and the continuous nature of the matrix phase versus the discrete distribution of grain-boundary precipitates, we estimate that Δ*ρ*_matrix_ accounts for approximately 70–90% of the total resistivity increase, with the remaining 10–30% attributed to grain-boundary silicides (Δ*ρ*_GB_). This analysis indicates that the matrix-phase modification through Al solid solution is the dominant factor in resistivity enhancement, while the grain-boundary silicides provide a supplementary contribution by impeding intergranular electron transport. This is consistent with the microstructural observation that Al is uniformly distributed throughout the matrix phase, whereas Si is localized at grain boundaries.

Based on the elemental distribution characteristics discussed in Section 3.1, the resistivity enhancement mechanisms can be understood as follows. In the matrix phase, the Al solid solution (~6 at. %) and dispersed Al(Tb,Dy)Fe_2_ nanoclusters collectively enhance electron scattering through lattice distortion and interface effects. At the grain boundaries, the Si-rich silicide phases (TbSi_1.75_ and Tb_2_Si_3_) act as resistive barriers that impede intergranular electron transport. The synergistic contribution from both matrix-level and grain-boundary-level modifications accounts for the substantial resistivity enhancement.

**Figure 7:**
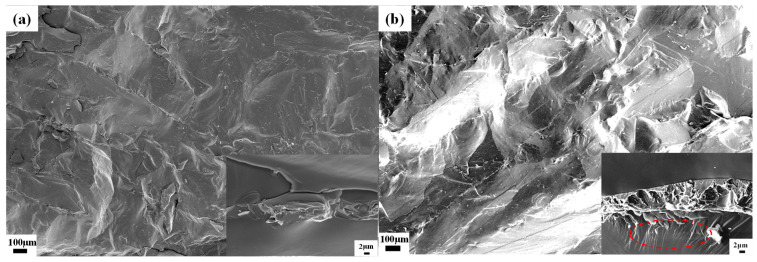
Fracture morphologies of directionally solidified <110>-oriented polycrystalline alloys: (**a**) TDF alloy, (**b**) TDF-AlSi alloy.

Comparative analysis of fracture morphologies is shown in Figure 7. From Figure 7a, it can be seen that the fracture surface of TDF alloy appears flat and smooth, exhibiting typical brittle fracture characteristics. The fracture surface exhibits a flat morphology with clear crack propagation paths, primarily characterized by intergranular fracture. In the directional solidified TDF alloy, grains align in parallel layers along the growth direction. This alignment causes cracks to propagate through grains with the same orientation over long distances, facilitating cleavage fracture. This fracture pattern suggests that the alloys experience quick brittle failure when subjected to external forces, showing minimal energy absorption and poor plastic deformation.

In contrast, although the TDF-AlSi alloy still exhibits brittle fracture behavior (Figure 7b), the fracture morphology displays notably more complex and rough characteristics compared to the unmodified alloy, indicating enhanced energy absorption during the fracture process. The high-magnification image (2 μm scale, lower right corner) reveals distinct microstructural features in different regions. In the grain boundary regions, numerous fine fibrous tensile traces and complex network-like microstructures are observed, including tearing ridges, step-like structures, and undulating features, which are characteristic of quasi-cleavage fracture with improved ductile response. These interlaced fibrous patterns suggest that crack propagation encountered significant resistance, requiring multiple deflections and bifurcations to bypass the strengthening phases. In the matrix region near the grain boundaries, a significant number of shear bands emerge (indicated by the red ellipse in Figure 7b), suggesting that the Al solid solution has modified the deformation behavior of the matrix, enabling localized plastic flow prior to final fracture. The above fracture characteristics correlate well with the observed 51.2% increase in bending strength (from 43 MPa to 65 MPa), which can be attributed to synergistic strengthening mechanisms. The addition of aluminum provides two strengthening effects: solid solution strengthening by distorting the lattice and precipitation strengthening through dispersed nanoscale Al(Tb,Dy)Fe_2_ precipitates in the matrix phase. Moreover, the formation of TbSi_1.75_ and Tb_2_Si_3_ phases at grain boundary regions not only enhanced boundary cohesion but also established an effective barrier network for crack propagation, compelling cracks to follow more tortuous paths. To clarify the relative contributions of matrix strengthening versus grain-boundary reinforcement, the following mechanistic analysis was performed. For matrix strengthening, the solid solution strengthening contribution from Al arises from atomic size misfit (~15% between Al and Fe) and modulus differences, which impede dislocation motion. With ~6 at. % Al uniformly distributed in the matrix, this mechanism is expected to contribute significantly to the overall strength increase. The Al(Tb,Dy)Fe_2_ nanoclusters (~10 nm, ~0.1–0.5 vol.%) provide additional precipitation strengthening by acting as obstacles to dislocation motion. For grain-boundary reinforcement, the silicide phases at grain boundaries (~8–12 vol.% grain boundary phase, ~40–50% of which contains silicides) primarily contribute to crack deflection and propagation resistance rather than initial yield strength. The fracture morphology (Figure 7b) provides direct evidence for these mechanisms: the shear bands in the matrix region indicate enhanced plasticity from Al solid solution, while the fibrous patterns at grain boundaries confirm effective crack deflection by silicides. Based on this analysis, we estimate that matrix strengthening (solid solution + precipitation) contributes approximately 60–70% of the total strength increase (~13–15 MPa), while grain-boundary reinforcement contributes approximately 30–40% (~7–9 MPa) primarily through improved crack propagation resistance and energy absorption during fracture. The complex fibrous patterns seen in the high-magnification images of Figure 7b directly reflect the coordinated deformation between the strengthening phases and the matrix phase. This demonstrates that the Al-Si co-addition effectively enhanced the mechanical properties of Tb-Dy-Fe alloys.

**Figure 8:**
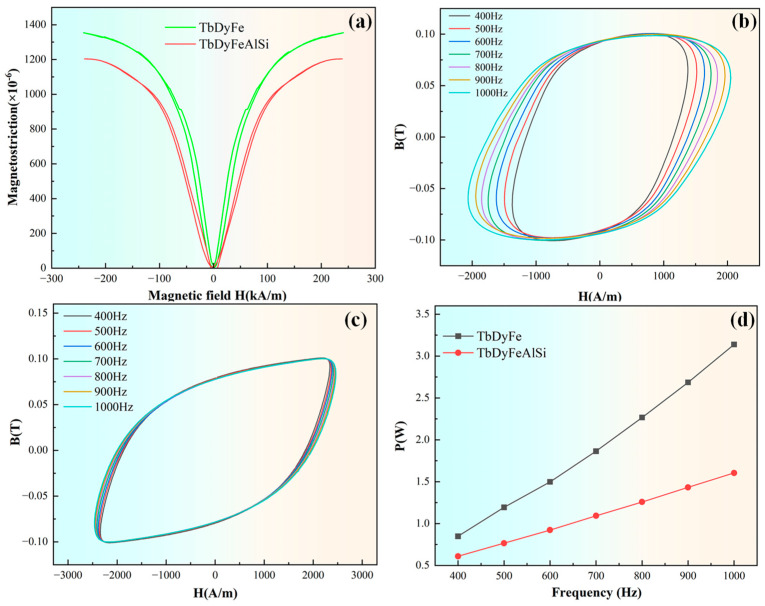
Magnetic properties of directionally solidified <110>-oriented polycrystalline TDF and TDF-AlSi alloys: (**a**) magnetostrictive curves of the two alloys under 10 MPa pre-compressive stress, (**b**) magnetic hysteresis loops of TDF alloy within the frequency range of 400–1000 Hz, (**c**) magnetic hysteresis loops of TDF-AlSi alloy within the frequency range of 400–1000 Hz, (**d**) comparison of total loss of the two alloys under different frequency.

The magnetostrictive behavior of directionally solidified polycrystalline specimens of TDF and TDF-AlSi was evaluated under 10 MPa axial pre-compression, with the measurement results presented in Figure 8a. At an applied field of 200 kA/m, the unmodified and Al-Si co-added alloys exhibited magnetostrictive strains of 1353 ppm and 1212 ppm, respectively. Despite a 10.4% reduction in strain output for the modified composition, the TDF-AlSi alloy still demonstrates substantial magnetostrictive capability, indicating that appropriate Al and Si incorporation preserves favorable magnetomechanical response. Comparable findings have been documented in prior investigations on single-element additions. Prajapati K et al. examined Fe-site substitution by Al in Tb_0.3_Dy_0.7_(Fe_1−x_Al_x_)_1.95_ (0 ≤ x ≤ 0.1) compositions and reported peak strain values approaching 1200 ppm when subjected to 120 kA/m field strength and 12 MPa compressive loading [21,28,29]. Similarly, Xu, L.H. et al. explored Si incorporation in Tb_0.3_Dy_0.7_(Fe_1−x_Si_x_)_1.95_ (x = 0, 0.025, 0.1) systems and observed that the x = 0.025 specimen achieved a maximum strain of 794 ppm under 4 kOe magnetic excitation in the stress-free condition [18].

The observed 10.4% decrease in magnetostriction can be attributed primarily to Al addition, with Si playing a secondary role. As established in Section 3.1, Al predominantly dissolves into the matrix phase while Si segregates to grain boundaries, resulting in fundamentally different influences on magnetostrictive behavior. For Al (primary factor), the incorporation of non-magnetic Al atoms into the (Tb,Dy)Fe^2^ matrix affects magnetostriction through four mechanisms: (1) reduced magnetic moment density due to replacement of magnetic Fe atoms, which weakens the overall magnetoelastic response; (2) altered local electronic structure that reduces the spin-orbit coupling strength—the fundamental microscopic origin of magnetostriction [21,27,28]; (3) modified magnetoelastic coupling coefficients, leading to reduced λ_111_ and λ_100_ values [27,28]; and (4) domain wall pinning by Al(Tb,Dy)Fe_2_ nanoclusters (~10 nm, Figure 4), which increases the energy barrier for domain wall motion and reduces low-field magnetostrictive response [30]. For Si (secondary factor), since Si preferentially segregates to grain boundaries forming TbSi_1.75_ and Tb_2_Si_3_ silicides, its direct impact on the matrix-dominated magnetostrictive response is minimal [18,30]. Potential indirect effects through modified grain boundary stress states, magnetic decoupling between grains, or local demagnetizing fields are expected to be secondary, given that the grain boundary phase occupies only ~8–12% of the total volume. This interpretation is supported by the observation that our magnetostriction reduction is comparable to values reported for similar Al substitution levels without Si addition [21].

The enhanced <110> preferred orientation observed after Al-Si co-addition (Figure 1b) may theoretically benefit magnetostrictive response by increasing the proportion of favorably oriented grains. However, the observed 10.4% decrease indicates that any beneficial texture effect is overwhelmed by the detrimental influence of Al substitution on intrinsic magnetoelastic coupling. Quantitative separation of texture and compositional effects would require future comparative studies with controlled texture development.

In summary, the distinct distribution behaviors of Al and Si—matrix dissolution versus grain boundary segregation—enable simultaneous optimization of multiple properties: significant improvements in mechanical strength (51.2%) and electrical resistivity (139.9%) are achieved with only a minor reduction in magnetostriction (10.4%), demonstrating effective synergistic performance enhancement.

Additionally, the enhanced <110> texture (Figure 1b) may contribute to the mechanical property improvement through anisotropic deformation behavior. The aligned columnar grain structure along the solidification direction could facilitate more uniform stress distribution, potentially contributing to the improved bending strength. However, quantitative separation of texture effects from compositional effects remains a direction for future investigation. fracture morphology.

The directionally solidified <110>-oriented polycrystalline TDF and TDF-AlSi specimens were examined by recording their dynamic hysteresis loops over a frequency span of 400–1000 Hz utilizing a NIM-3000S soft magnetic measurement system, with the outcomes illustrated in Figure 8b–d. The enclosed region within each hysteresis loop quantifies the energy consumption during one complete magnetization-demagnetization cycle. By integrating the measured loop area, the total core loss at a given operating frequency can be obtained. Figure 8b,c display the dynamic hysteresis responses of both alloys across the 400–1000 Hz frequency range. The AC magnetization data reveal that the TDF-AlSi alloy exhibits diminished core loss following Al and Si co-incorporation. The frequency-dependent loss characteristics derived from loop integration are depicted in Figure 8d, with corresponding numerical data tabulated in Table 3. These results demonstrate that the aggregate loss of the modified alloy decreases substantially after Al and Si co-addition. As evidenced in Table 3, the loss reduction exhibits a positive correlation with operating frequency, escalating from 28.2% at 400 Hz to 49.0% at 1000 Hz. This trend is primarily ascribed to the elevated electrical resistivity of the modified alloy. The observed frequency-dependent behavior aligns well with the theoretical quadratic relationship between eddy current loss and excitation frequency.

It should be noted that our analysis of AC magnetic loss reduction is based primarily on the eddy current loss mechanism, which exhibits the characteristic quadratic frequency dependence (P_eddy_ ∝ f^2^/*ρ*) as observed in Figure 8d. This interpretation implicitly assumes that changes in intrinsic magnetic damping (viscous domain wall motion) and quasi-static hysteresis loss are secondary to the eddy current contribution. This assumption is reasonable for the following reasons: (1) The total loss scales approximately with f^2^ as expected for eddy-current-dominated behavior; (2) The 139.9% increase in resistivity directly corresponds to the observed loss reduction trend; (3) The Al-Si co-addition does not significantly alter the coercivity or hysteresis loop shape at low frequencies (Figure 8b,c), suggesting minimal change in quasi-static hysteresis loss.

## 4. Conclusions

The effects of Al-Si co-addition on the microstructure and properties of directionally solidified <110>-oriented Tb_0.27_Dy_0.73_Fe_1.95_ alloys were systematically investigated. Microstructural analyses revealed distinct distribution behaviors: Al predominantly dissolved into the matrix phase forming substitutional solid solutions and nanoscale Al(Tb,Dy)Fe_2_ clusters (~10 nm), whereas Si preferentially segregated to grain boundaries forming Tb_2_Si_3_ and TbSi_1.75_ silicides.

The Al-Si co-addition strategy achieved significant improvements in electrical resistivity and mechanical properties while maintaining acceptable magnetostrictive performance. Specifically, the electrical resistivity increased by 139.9% (from 0.675 to 1.619 μΩ·m), resulting in a 49% reduction in core loss at 1000 Hz. The bending strength improved by 51.2% (from 43 to 65 MPa). Meanwhile, the magnetostrictive strain remained at 1212 ppm (200 kA/m, 10 MPa), showing only a 10.4% decrease compared to the unmodified alloy.

The enhanced electrical resistivity is attributed to lattice distortion from Al substitution combined with electron impedance at silicide-containing grain boundaries. The mechanical improvement results from synergistic strengthening: solid-solution and precipitation strengthening in the matrix, coupled with crack deflection by grain boundary silicides.

This work demonstrates that dual-phase modification through Al-Si co-addition represents a promising approach for comprehensive performance optimization of Tb-Dy-Fe alloys. Future studies should explore the Al-Si composition space to establish general design guidelines, and characterize high-frequency (10–50 kHz) and temperature-dependent properties for practical device applications.

## Figures and Tables

**Table 1:** Chemical compositions of TDF and TDF-AlSi alloy (shown in Figure 2b,d) determined by energy-dispersive X-ray spectroscopy (EDS) (at. %).

Points	Chemical Compositions (at. %)				
	Fe	Tb	Dy	Al	Si
1	65.39 ± 0.89	11.17 ± 0.53	23.54 ± 0.67	0.00	0.00
2	0.00	43.12 ± 0.93	56.88 ± 0.88	0.00	0.00
3	0.00	28.47 ± 0.97	46.33 ± 1.11	0.00	25.20 ± 0.07
4	0.00	24.93 ± 0.97	43.78 ± 1.12	0.64 ± 0.06	30.65 ± 0.08
5	56.73 ± 0.51	12.09 ± 0.65	24.01 ± 1.20	6.13 ± 0.05	1.04 ± 0.03

**Table 2:** TEM-EDS elemental composition analysis of the region A shown in Figure 4a in TDF-AlSi alloy (at. %).

Element	Atom%
Al	21.95 ± 0.34
Fe	39.96 ± 0.43
Tb	15.97 ± 0.41
Dy	22.12 ± 0.62

**Table 3:** Core loss values of TDF and TDF-AlSi alloys at different frequencies.

Frequency (Hz)	TDF (W)	TDF-AlSi (W)	Reduction (%)
400	0.85	0.61	28.2
500	1.20	0.76	36.7
600	1.50	0.92	38.7
700	1.86	1.09	41.4
800	2.26	1.25	44.7
900	2.70	1.43	47.0
1000	3.14	1.60	49.0

Note: The total loss (W) was calculated by multiplying the specific loss (W/kg) by the sample mass.

## Data Availability

The original contributions presented in the study are included in the article. Further inquiries can be directed to the corresponding author.
